# Mind-Body Therapies: Evidence and Implications in Advanced Oncology Practice

**Published:** 2012-11-01

**Authors:** Kelley D. Mayden,

**Affiliations:** From Southwest Virginia Cancer Center, Norton, Virginia

## Abstract

The idea that thoughts and emotions influence health outcomes is an ancient concept that was initially abandoned by Western medicine researchers. Today, researchers are showing a renewed interest in the interactions of the mind and body and the role these interactions play in disease formation and recovery. Complementary and alternative interventions, such as mind-body therapies, are increasingly being used by cancer survivors for disease prevention, immune system enhancement, and symptom control. Traditional training has not been structured to provide advanced practitioners with an in-depth knowledge of the clinical applications of mind-body therapies. The aim of this article is to acquaint the reader with common mind-body modalities (meditation/mindfulness-based stress reduction, relaxation therapy, cognitive-behavioral therapy, hypnosis, biofeedback, music therapy, art therapy, support groups, and aromatherapy) and to examine important evidence in support of or against their clinical application.

The earliest recordings of human existence document that every society has been besieged by various maladies. Yet despite varying belief systems, the common goal has always been to restore the body to a state of wholeness. Medical models from ancient Egypt, Babylon, and Greece all incorporated treatment of the body as well as the mind. Likewise, both traditional Chinese medicine and Indian ayurvedic medicine contain elements that allow for the treatment of both. Central to these systems is the belief that interactions between the mind and body can bring about disease and affect health and healing. This is in contrast to modern Western medicine (MWM), which traditionally has focused on the body, evidence, and drug therapy (Verkerk, 2009).

Although it is not entirely obvious at what point the focus shifted or who crowns the MWM genogram, it likely traces back to the microscopic observation of bacteria and microorganisms in 1676 by Antonie van Leeuwenhoek. In 1700, Nicolas Andry theorized that smallpox resulted from bacterial invasion. Although poorly received at first, the germ theory would later be validated by French chemist Louis Pasteur. Building on van Leeuwenhoek’s observations, Pasteur provided evidence that disease was a direct result of invasion by microorganisms. In 1862, Pasteur, along with Claude Bernard, developed the process of pasteurization, which is still in use. Other innovators of the 19th century include Gregor Mendel, Joseph Lister, and Robert Koch. In 1852, Florence Nightingale, who established St. Thomas Hospital in London, became a noted champion of hygiene and nutrition, leading to a measurable reduction in mortality.

Building on the established roots of these notable contributors, the 20th century would define medicine’s paradigm as one underwritten by the scientific method. Through pandemics, epidemics, and various wars, technology has continued to advance and utilization of the scientific method has brought us full circle from discovery of the structure of DNA by Watson and Crick (1953) to international sequencing of the human genome. While such unprecedented achievements leave little room for criticism, they support an operational definition of illness as an entity of external cause with internal effect, driving modern science to focus on the development of externally administered treatments that control symptoms or cure disease.

More recently, building on the groundbreaking observations of modern mind-body (MB) medicine researchers such as Walter Bradford Cannon and Hans Selye, MWM has demonstrated a renewed interest in the interactions of the mind and body and what role these interactions play in disease formation and recovery. The investigations of George Solomon, Robert Ader, Claus Bahnson, Caroline Thomas, and others cumulatively suggest that disease states, including cancer, are a manifestation of not only external invaders, but are influenced by internal factors such as immunity and emotions (Gordon, 2008). Currently, there is at least partial acceptance from most of the scientific community that indeed there is a MB connection and that it is related to illness and health.

In true Western fashion, researchers across all disciplines are applying the scientific method to map the interactions of the mind, nervous system, immune system, and the body. Devoted to untangling the MB mystery, psychoneuroimmunology is a rapidly expanding field that seeks to determine the relationship between psychological processes and health (Lorentz, 2006). In the future, it is likely that the links between mental processes, disease, and symptom manifestations will be more fully understood and that therapeutic interventions will fall outside of traditional pharmaceuticals.

In order to help clinicians develop a better understanding of nontraditional medicine and the MB connection, some academic institutions are incorporating complementary and alternative medicine (CAM) into their curriculum; however, this has not traditionally been the case. This has left medical professionals—including physicians, medical students, advanced practitioners, and nurses—with limited knowledge and inadequate training in the use of CAM (Lee, Hlubocky, Hu, Stafford, & Daugherty, 2008; Yildirim et al., 2010). Meanwhile, individuals with illnesses who suffer MB symptoms such as anxiety, fear, depression, pain, fatigue, decreased quality of life (QOL), immune dysfunction, and other symptoms, including those with cancer, are embracing CAM. Recent analysis of data from the 2007 National Health Interview Survey found that among cancer survivors, 66.5% reported ever using CAM and 43.3% reported using CAM within the past year. Compared with the general population, cancer survivors used CAM more often for general disease prevention, immune system enhancement, and pain control (Mao, Palmer, Healy, Desai, & Amsterdam, 2011).

The National Center for Complementary and Alternative Medicine (NCCAM) defines CAM as a group of diverse medical and health care systems, practices, and products that are not generally considered part of conventional medicine. Further, NCCAM has grouped CAM practices into broad categories, such as natural products, mind-body medicine, and manipulative and body-based practices (NCCAM, 2012). Since 2004, the National Cancer Institute has invested more than $120 million annually in cancer CAM research; in 2009, 5.5% of funding was directed toward MB interventions (Jia, 2012).

Given the growing interest in MB therapies on the part of providers and patients, this article will review important findings with regard to MB therapies in the oncology setting and attempt to assist providers with complementary integration at the bedside. It is important to understand that MB modalities are true complementary interventions (those combined with standard therapy) and not alternative therapies. By definition, alternative therapies have not been validated but are often substituted for standard therapy and may pose risk or harm to patients (Deng et al., 2009). Recommended organizations and websites for additional information on the benefits and risks of CAM are outlined in Table 1.

**Table 1:**
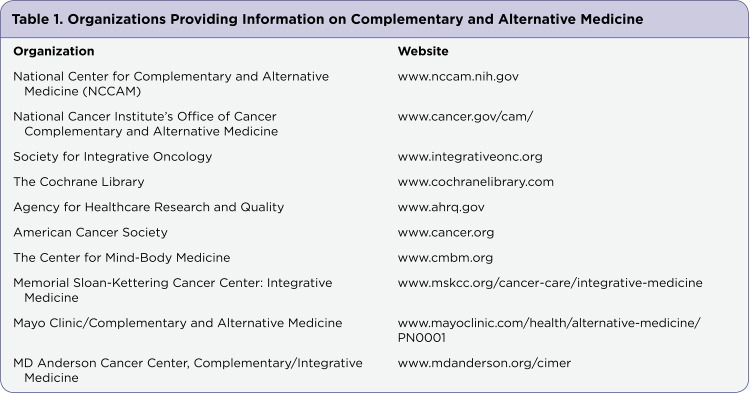
Table 1. Organizations Providing Information on Complementary and Alternative Medicine

## Mind-Body Interventions

Mind-body interventions are practices that focus on the interactions among the brain, body, mind, and behavior with the intent of using the mind to alter physical function and promote overall health (Elkins, Fisher, & Johnson, 2010). Mind-body approaches should be part of a multidisciplinary approach and aimed at reducing anxiety, mood disturbance, pain and improving QOL (Deng et al., 2009). Understandably, a diagnosis of cancer can generate feelings of helplessness and hopelessness and is associated with a myriad of psychosocial difficulties. Understanding and assisting patients with appropriate MB interventions can enhance the provider-patient relationship, improve symptoms, and enable patients to assign meaning to their experience.

**MEDITATION/MINDFULNESS-BASED STRESS REDUCTION** 

Meditation, adapted from traditional Eastern systems, focuses attention on increasing mental awareness and clarity of mind (concentrative meditation) or opens attention to thoughts, feelings, and sensations that go through the mind from moment to moment (mindfulness meditation; Deng et al., 2009; Gordon, 2008). Data in the oncology setting are largely from studies that explored a form of meditation known as mindfulness-based stress reduction (MBSR), which incorporates acceptance, mediation, yoga, stretching, and group dynamics and is modeled after the MBSR program of Jon Kabat-Zinn and colleagues at the University of Massachusetts Medical Center (Carlson, Speca, Patel, & Goodey, 2003; Deng et al., 2009).

A randomized, wait-list, controlled trial assessing the effects of participation in a MBSR program on mood disturbance and symptoms of stress in 91 cancer outpatients (mean age, 51 years) found that those who participated in a weekly meditation group lasting 1.5 hours for 7 weeks plus home meditation practice experienced a 65% decrease in mood disturbance and a 31% reduction in stress symptoms, in both male and female patients with a wide variety of cancer diagnosis, stage of illness, and age as compared to those randomized to immediate treatment only (Speca, Carlson, Goodey, & Angen, 2000). Upon completion of the program, 54 patients provided postintervention data indicating that positive results were maintained at 6 months (Carlson, Ursuliak, Goodey, Angen, & Speca, 2001). A group of early-stage breast and prostate cancer patients (N = 59) who participated in a MBSR program delivering eight weekly, 90-minute group sessions and a 3-hour silent retreat on the Saturday between weeks 6 and 7 experienced significant improvement in overall QOL, symptoms of stress, and sleep quality. The total number of sleep hours increased by one-half hour, but this was not statistically significant. Additionally, T-cell production of interleukin (IL)-4 increased and interferon (IFN)-ã decreased, whereas natural killer (NK) cell production of IL-10 decreased. Small sample size and the lack of a comparison group were the major methodologic limitations of this study (Carlson et al., 2003).

Mindfulness-based stress reduction in relation to QOL; mood; symptoms of stress; and levels of cortisol, dehydroepiandrosterone sulfate (DHEAS), and melatonin was evaluated in a group of early-stage breast cancer (n = 59) and prostate cancer (n = 10) patients. There was significant improvement in overall QOL (* p* < .05), symptoms of stress (* p* < .01), and sleep, but the improvements did not significantly correlate with the degree of program attendance or minutes of home practice. Although DHEAS and melatonin changes were not observed, results pointed to possible beneficial changes in hypothalamic-pituitary-adrenal axis functioning by documented attenuation of elevated diurnal cortisol secretion (Carlson, Speca, Patel, & Goodey, 2004). A 1-year pre/post intervention follow-up of psychological, immune, endocrine, and blood pressure outcomes of MBSR in breast and prostate cancer outpatients concluded that the program was associated with enhanced QOL, decreased stress symptoms, and altered cortisol and immune patterns consistent with less stress and mood disturbance and decreased blood pressure (Carlson, Speca, Faris, & Patel, 2007).

In a nonrandomized controlled trial of women with newly diagnosed and early-stage breast cancer, the effects of MBSR on immune function, QOL, and coping were assessed after an 8-week program. The MBSR group reestablished their NK cells and cytokine production levels and had reduced cortisol levels, improved QOL, and increased coping effectiveness compared to the non-MBSR cohort (Witek-Janusek et al., 2008). In a trial of 229 women with stage 0 to III breast cancer treated with surgery, chemotherapy, and radiotherapy and evaluated for the effectiveness of MBSR on mood, breast, and endocrine-specific QOL and well-being, results confirmed MBSR improved mood, breast, and endocrine-related QOL and well-being more effectively than standard care. These results persisted at 3 months (Hoffman et al., 2012). A 10-week MBSR program was shown to reduce persistent fatigue in a group of breast cancer survivors (N = 68) at least 6 months postadjuvant chemotherapy or radiation therapy with a baseline fatigue score of 50 or lower on the vitality and fatigue subscale of the SF-36 Health Survey. Compared to baseline, SF-36 scores increased by 60% (* p* < .001). Likewise, fatigue as measured by the Piper Fatigue Scale and visual analog scale pre/post intervention decreased by 40% (* p* < .001; Appling, Scarvalone, MacDonald, McBeth, & Helzlsouer, 2012).

Results from a recent meta-analysis examining the evidence for MBSR in cancer care reported significant improvements in anxiety, depression, stress, sexual difficulties, physiologic arousal, and immune function across all interventions. Methodologic limitations were identified, and, among various studies, diversity in study design and intervention made comparison between studies difficult (Shennan, Payne, & Fenlon, 2011). Two additional meta-analyses, one examining MBSR among breast cancer survivors and one Norway-based review, suggest improvements in stress symptoms, mood disturbance, and improved QOL in cancer patients (Matchim, Armer, & Stewart, 2011; Musial, Bussing, Heusser, Choi, & Ostermann, 2011).

**RELAXATION THERAPY** 

The use of relaxation therapy dates back to the early 1900s and was popularized with the printing of *The Relaxation Response* in 1975 (Benson & Klipper, 1975). Since then, relaxation therapies have been incorporated into MB interventions. Relation therapies are measures designed to produce a state of relative freedom from mental and/or physical tension. It is theorized that relaxation therapies minimize sympathetic nervous system response, which in turn decreases oxygen demand, slows heart rate, and lowers blood pressure (Elkins et al., 2010). Relaxation therapies may incorporate a variety of techniques such as deep breathing, guided imagery, progressive relaxation, meditation, yoga, self-hypnosis, and biofeedback (NCCAM, 2012).

Early evidence suggested that in 26 cancer subjects who received muscle relaxation training, mean sleep onset latency was reduced for the majority of participants. In 15 patients, sleep onset latency was reduced from 124 to 29 minutes (Cannici, Malcolm, & Peek, 1983). A randomized controlled trial comparing alprazolam to progressive muscle relaxation (PMR) concluded that both treatment arms resulted in a significant (* p* < .001) decrease in observer- and patient-reported anxiety and depressed mood symptoms. Although both treatment arms were effective, patients receiving the drug showed a slightly more rapid decrease in anxiety and greater reduction in depressive symptoms (Holland et al., 1991).

In another trial, 82 outpatients undergoing curative or palliative radiotherapy were randomly assigned to relaxation training or no relaxation training during their course of treatment. Analysis revealed that the addition of relaxation training decreased symptoms of tension, depression, anger, and fatigue (* p* < .01) when pre/post Profile of Mood States assessments were compared (Decker, Cline-Elsen, & Gallagher, 1992).

In a review of data from interviews conducted among cancer patients who underwent a trial of PMR and guided imagery, interventions were designed to analyze patient perceptions of treatment effects with observed changes in pain scores; 81% (21 patients) reported that PMR worked to relieve their pain, but only 10 patients were also categorized as responders based on observed pain scores. Overall perceptions of the effectiveness of PMR matched observed changes in pain 54% of the time (Kwekkeboom, Hau, Wanta, & Bumpus, 2008).

Among 49 women with newly diagnosed breast cancer undergoing adjuvant therapy, relaxation practices (progressive, imagination, deep breathing, and visualization) were assessed, along with immune measures, for 10 months. The mean overall extent of therapy was 106.5 minutes per week. Relaxation practices significantly contributed to the variance of NK cell activity, lymphocyte proliferation, and IL-4 and IL-10 responses in a positive direction; IL-2 and IL-6 responses were not affected. Longer durations of relaxation practice were associated with increases in all immune measures, higher-stage cancer was associated with increases in IL-2, and combination therapy was associated with increased IL-6. The majority of participants preferred deep breathing (65%); the second most commonly chosen method was progressive relaxation (12%; Kang et al., 2011).

**COGNITIVE-BEHAVIORAL THERAPY** 

Coined by Aaron Beck in the 1960s, the term cognitive-behavioral therapy (CBT) refers to interventions aimed at altering a patient’s thoughts, behaviors, or emotional responses to assist in recognizing and controlling response to symptoms using a programmed education or counseling approach (Kwekkeboom, Cherwin, Lee, & Wanta, 2010). It can involve relaxation or guided imagery, in which the patient uses his or her imagination to create mental images that distract attention away from symptoms (Kwekkeboom et al., 2008).

Analysis of 21 CBT-based studies involving patients with cancer-related pain, fatigue, or sleep disturbance demonstrated that CBT interventions were efficacious in 14 of 21 of them. The studies demonstrated improvements in all three symptoms, but the majority of support favored fatigue and sleep disturbance (Kwekkeboom et al., 2010). Mindfulness-based CBT involving 8 weekly 2-hour sessions was compared to treatment alone in 115 patients with cancer across site and stage. The CBT group had significant improvements in mindfulness (effect size [ES] = 0.55), depression (ES = 0.83), anxiety (ES = 0.59), and distress (ES = 0.53), with a trend toward improvement in QOL (ES = 0.30; Foley, Baillie, Huxter, Price, & Sinclair, 2010).

In an attempt to assess initial efficacy of a patient-controlled CBT intervention for pain, fatigue, and sleep, 86 patients with lung, prostate, colorectal, and gynecologic cancers were randomly assigned to training in and use of up to 12 CBT exercises delivered via an MP3 player for 2 weeks during cancer treatment or control. Outcomes included symptom cluster severity and overall symptom interference with daily life. Follow-up at 2 weeks found symptom cluster severity lower in the intervention group. Symptom interference with daily life did not differ between groups. No adverse events were associated with the CBT interventions (Kwekkeboom et al., 2012). In a systematic review of CBT interventions in advanced cancer, with 11 studies meeting inclusion criteria, Campbell and Campbell found that interpretation of the effectiveness of CBT interventions was limited by major challenges to the internal validity of the studies included in the review. They concluded that the lack of data regarding the efficacy of CBT interventions in supporting individuals with advanced cancer demonstrates a gap in the current knowledge base (Campbell & Campbell, 2012).

**HYPNOSIS** 

Introduced in the 18th century by Franz Anton Mesmer, hypnosis has been extensively researched in a number of settings. Hypnosis is defined as an agreement between a person designated as the health-care hypnotist and a person designated as the patient to participate in a psychotherapeutic technique in which the hypnotist provides suggestions for changes in sensation, perception, cognition, affect, mood, or behavior (Montgomery et al., 2010). Hypnosis has documented efficacy in a variety of conditions such as mental health disorders, smoking cessation, obesity, pain reduction, anxiety, and nausea and vomiting (Deng et al., 2009; Elkins et al., 2010; Monti, Sufian, & Peterson, 2008). Optimal results are achieved by a trained therapist who has developed a good rapport and a strong level of trust with the patient. A comfortable environment, free of distraction, is needed, and the patient must be willing to undergo the procedure (Cassileth et al., 2007).

Early studies support hypnosis as a means to control pain associated with breast cancer, reduce chemotherapy distress in children, decrease anticipatory nausea and vomiting and antiemetic use in children receiving chemotherapy, decrease reported oral pain related to bone marrow transplantation, and decrease pain and pain-related anxiety in pediatric patients undergoing bone marrow aspirations (Jacknow, Tschann, Link, & Boyce, 1994; Liossi & Hatira, 1999; Morrow & Morrell, 1982; Spiegel & Bloom, 1983; Syrjala, Cummings, & Donaldson, 1992; Zeltzer, Dolgin, LeBaron, & LeBaron, 1991). In 1996, a National Institutes of Health (NIH) Technology Assessment Panel determined that hypnosis alleviated cancer-related pain (NIH Technology Assessment Panel, 1996).

In a noncancer setting, hypnosis has been shown to reduce the discomfort associated with invasive medical procedures (Lang et al., 2000). In 2002, hypnosis reduced postsurgery pain and distress in a group of 20 patients undergoing excisional breast biopsy (Montgomery, Weltz, Seltz, & Bovbjerg, 2002). In a randomized controlled trial of 39 advanced-stage cancer patients with malignant bone disease comparing hypnosis with supportive attention as a means to control pain, hypnosis demonstrated a statistically significant decrease in pain (Elkins, Cheung, Marcus, Palamara, & Rajab, 2004).

More recently, in a group of 236 women referred for large core needle breast biopsy, randomized to standard care, structured empathic attention, or adjunct self-hypnotic relaxation as a means to control pain and reduce anxiety associated with the procedure, patients in the hypnosis arm experienced decreased anxiety as compared to those in the other arms (* p* < .001). Pain significantly increased in all three groups, though less steeply with the hypnosis and empathy groups. The study suggests that hypnosis is more effective for anxiety relief (Lang et al., 2006).

In a separate study, 200 patients scheduled to undergo excisional breast biopsy or lumpectomy were randomly assigned to a 15-minute presurgery hypnosis session or nondirective empathic listening (attention control). Intraoperative anesthesia use (lidocaine, fentanyl, propofol, and midazolam) was assessed. Patients in the hypnosis group required less propofol (mean difference 32.63 ìg) and lidocaine (mean difference 6.86 mL). Patients in the hypnosis group also reported less pain intensity, fatigue, discomfort, and emotional disturbance with a collectively significant effect (* p* < .0001). No statistically significant differences were seen in the use of fentanyl, midazolam, or recovery room analgesics (Montgomery et al., 2007).

Vasomotor syndrome (hot flashes) is an uncomfortable yet frequent clinical complaint elicited from women dealing with breast cancer and cancer treatment. Often, it is a direct result of the treatment itself. Given that hormonal replacement in this population is taboo and the limited effective pharmacologic options, MB interventions such as hypnosis are in ongoing investigation.

One study from 2008 conducted among 60 female breast cancer survivors with hot flashes evaluated the effect of 5 weekly hypnosis sessions vs. no treatment (Elkins et al., 2008). By the end of the treatment period, hot flash scores decreased 68% from baseline in the hypnosis group (* p* < .001). Significant improvements in self-reported anxiety, depression, interference of hot flashes on daily activities, and sleep were observed for patients who received the hypnosis intervention. Satisfaction with the hypnosis intervention was favorable, with no report of adverse effects, suggesting hypnosis as a feasible treatment option that deserves additional investigation (Elkins et al., 2008). At the time of publication, a larger trial (NCT01293695) is recruiting 180 postmenopausal women who will be randomly assigned to hypnosis vs. structured attention control as a means to study the perceived impact and physiologically measured impact of hypnosis on hot flashes.

Breast cancer radiotherapy and tumor radiofrequency can cause pain, anxiety, or mood disturbances. Hypnosis has been explored as a means to improve patient tolerance to both procedures. For their tumor embolization or radiofrequency ablation, 201 patients were randomly assigned to receive standard care, empathic attention, or self-hypnotic relaxation delivered at the time of procedure. The main intent was to evaluate the effects of each on pain and anxiety. Hypnosis patients experienced significantly less pain and anxiety than those receiving standard care, and empathy patients received less median drug units, thus indicating that procedural hypnosis (including empathic attention) reduces pain, anxiety, and medication use (Lang et al., 2008).

In another study, women undergoing breast cancer radiotherapy (N = 40) who received a 15-minute hypnosis session combined with CBT on the day of their simulation, prior to the simulation, experienced reduced levels of negative affect and increased levels of positive affect as compared to those who received standard care. Participants then completed weekly self-report measures of positive and negative affect. The CBT group had a significantly higher frequency of days where positive affect was greater than negative affect (85% of days for CBT vs. 43% for control; Schnur et al., 2009).

Despite the extent to which hypnosis has been investigated, a systematic review of hypnotherapy for treating symptoms in terminally ill adult cancer patients concluded that in this population, the number and quality of studies were poor, and further research was needed to determine the effects of hypnosis for symptom management in terminal populations (Rajasekaran, Edmonds, & Higginson, 2005). Recently, a pilot study conducted in a hospice setting in Surrey, England, evaluated the effects of four sessions of hypnotherapy in the management of anxiety and other symptoms including depression and sleep disturbance. Data from 11 patients completing intervention suggested that after the second session, there was a statistically significant reduction in mean anxiety and symptom severity, but not in depression or sleep disturbance. After the fourth session, significant reduction in all symptoms was reported (Plaskota et al., 2012).

**BIOFEEDBACK** 

Originally explored by Neal Miller, biofeedback (BF) is a process that enables an individual to learn how to change physiologic activity for the purposes of improving health and performance. Validated instruments measure physiologic activity such as brainwaves, heart function, breathing, muscle activity, and skin temperature. These instruments then accurately provide feedback to the user. The presentation of this information, often in conjunction with changes in thinking, emotions, and behavior, supports desired physiologic changes. Over time, these changes can persist without use of an instrument (Association for Applied Psychophysiology and Biofeedback, 2008). In the 1990s, data specific to BF in the cancer setting were limited and suggested that alone, BF was of minimal benefit in reducing the side effects associated with cancer chemotherapy (Burish & Jenkins, 1992).

In 2005, 25 breast cancer patients who had completed adjuvant chemotherapy were asked to participate in a study to determine the effects of abdominal breathing training using BF on stress, immune response, and QOL. After four weekly training sessions, anxiety, cancer physical symptoms, and serum cortisol were reduced, but there was no statistically significant difference from the control group. However, QOL was improved in the trained group (Kim et al., 2005). A small group of patients with advanced cancer in a palliative care unit receiving BF-assisted relaxation experienced a reduction in cancer-related pain compared to those receiving conventional care (Tsai, Chen, Lai, Lee, & Lin, 2007).

There is evidence to suggest that BF, coupled with behavioral and pelvic muscle training, is effective for improving time to continence, decreasing incontinence severity, and trends to an improvement in QOL after prostatectomy (Burgio et al., 2006; Tienforti et al., 2012). Similarly, patients undergoing bowel resection for colorectal cancer have reported improvement in fecal incontinence scores, number of bowel movements, and improved fecal incontinence QOL scores. Improvement was demonstrated even if BF was started 18 months after surgery (Bartlett, Sloots, Nowak, & Ho, 2011; Kim et al., 2011).

Management of respiratory motion, a common concern during radiation therapy, has led to the development of respiratory-gated treatment techniques to limit organ motion. A protocol consisting of five breathing training sessions allowing for free breathing, breathing with audio instructions, and breathing with audio-visual biofeedback (AVBF) was evaluated in 24 lung cancer patients to determine if BF would improve respiratory reproducibility by decreasing residual motion and increasing the accuracy of gated radiotherapy. Use of AVBF significantly reduced residual motion compared with the other training methods (George et al., 2006). A second study of 12 non–small cell lung cancer (NSCLC) patients comparing free breathing with video biofeedback trained breathing (VBTB) during radiation therapy concluded that VBTB appeared to be superior to free breathing in terms of control over reproducibility of baseline amplitudes and frequencies and could produce stable breathing patterns for patients during radiation therapy for NSCLC (Al-Mohammed, 2010).

**MUSIC THERAPY** 

The writings of Aristotle and Plato suggest that music was an important part of education, as well as religious and civil life, in ancient times. It was believed that music was allied with the intricacies of the body and mind and therapeutic in and of itself. Currently, music therapy (MT) is recognized as the "clinical and evidence-based use of music interventions to accomplish individualized goals within a therapeutic relationship by a credentialed professional who has completed an approved music therapy program" (American Music Therapy Association, 2012). Interventions are designed to promote wellness, manage stress, alleviate pain, express feelings, enhance memory, improve communication, and promote physical rehabilitation (American Music Therapy Association, 2012). Extensively studied in the acute care setting, MT has application in the oncology setting and is becoming an increasingly popular adjunctive intervention for supporting the psychosocial needs of cancer survivors (Monti, Sufian, & Peterson, 2008).

In a survey examining the extent to which cancer patients use certain coping strategies, music was the most commonly utilized coping method next to prayer (Zaza, Sellick, & Hillier, 2005). Music therapy has been shown to decrease anxiety associated with chemotherapy and breast biopsy (Sabo & Michael, 1996; Haun, Mainous, & Looney, 2001). An important study examining the effects of MT on QOL and length of life in a terminal population revealed a significant improvement in QOL for those in the MT group as measured by the Hospice Quality of Life Index–Revised. Those in the control group (without MT) experienced a decreased QOL over time (Hilliard, 2003). Patients undergoing autologous stem cell transplantation (n = 69) experienced less total mood disturbance when they received MT during inpatient stays. Of the 62 patients available for evaluation, patients in the MT group scored 28% lower on the combined Anxiety/Depression scale (*p* = .065) and 37% lower on the total mood disturbance score (*p* = .01) compared to controls (Cassileth, Vickers, & Magill, 2003).

In a palliative setting of 200 patients with advanced disease, MT improved body movement, facial expression, mood, pain, shortness of breath, and verbalizations, while also reducing anxiety. Family members were involved as well, and they experienced an improvement in facial expressions, mood, and verbalizations (Gallagher, Lagman, Walsh, Davis, & Legrand, 2006). A quantitative study evaluating the effect of a single MT session on anxiety as measured by the Edmonton Symptom Assessment System in hospitalized end-stage patients found a significant reduction in anxiety (*p* = .005) in the MT group. A post hoc analysis found significant reductions in pain (*p* = .019), tiredness (*p* = .024), and drowsiness (*p* = .018) in the MT group (Horne-Thompson & Grocke, 2008).

During lumbar puncture, children assigned to a MT group experienced lower pain scores and lower heart and respiratory rates during and after the procedure (Nguyen, Nilsson, Hellstrom, & Bengtson, 2010).

Women with breast and cervical cancer receiving chemotherapy who received 20 minutes of MT twice a day for 3 days experienced a decrease in pain and increase in relaxation (Kaliyaperumal & Subash, 2010). Additional trials support the idea that the addition of MT to the chemotherapy setting lowers anxiety, decreases fear, lowers heart rate, and promotes relaxation, particularly among individuals with high levels of anxiety (Ferrer, 2007; Lin, Hsieh, Hsu, Fetzer, & Hsu, 2011). Although physical symptoms were not affected by the addition of MT in patients undergoing curative radiation therapy, treatment-related distress was decreased in those who received MT (Clark et al., 2006).

Women undergoing radical mastectomy experience many procedural-related symptoms, including pain. In a group of 120 breast cancer patients receiving personal controlled analgesia postsurgery, half were randomized to MT from the first day after surgery to the third admission for chemotherapy. In the MT group, pain rating index scores fell and pain intensity improved. These findings were consistent across three posttests and as measured by Pain Rating Index and Present Pain Intensity scores (Li et al., 2011). In a separate study, women treated with MT after radical mastectomy demonstrated significant improvements in depression (* p* < .001) as measured by the General Questionnaire and the Chinese version of the Zung Self-Rating Depression Scale and a decrease in the length of hospital stay (t = -4.34, * p* < .001) as compared to women who received routine nursing care (Zhou, Li, Yan, Dang, & Wang, 2011).

Results from a trial of 70 women with metastatic breast cancer designed to examine the effects of MT immediately and over time on patients’ psychological functioning, QOL, and stress arousal found that delivery of three MT sessions led by a music therapist resulted in significant immediate effects on patient relaxation (* p* < .00001), comfort (* p* < .00001), happiness (* p* < .00001), and heart rate (*p* = .0003); over time, no significant differences were observed between the MT group and the control group. A limiting factor may have been a high attrition rate (Hanser et al., 2006). A meta-analysis of 30 trials with a total of 1,891 participants indicated that MT may have beneficial effects on anxiety, pain, mood, and QOL in people with cancer. Notably, most trials were at high risk of bias (Bradt, Dileo, Grocke, & Magill, 2011).

**ART THERAPY** 

"Art therapy (AT) is the therapeutic use of art making, within a professional relationship, by people who experience illness, trauma, or challenges in living, and by people who seek personal development. Through creating art and reflecting on the art products and processes, people can increase awareness of self and others; cope with symptoms, stress, and traumatic experiences; enhance cognitive abilities; and enjoy the life-affirming pleasures of making art" (American Art Therapy Association, 2012). Conducted by a trained art therapist, usually a Master’s level professional, art therapy has demonstrated benefits in patients with cancer. It is considered a safe complementary therapy.

In the chemotherapy setting, 51 of 54 patients perceived AT as helpful for expressing emotions and meeting needs (Forzoni et al., 2010). Pediatric brain tumor patients receiving outpatient chemotherapy randomized to creative arts therapy showed improved mood, and were more excited, happier, and less nervous than those who received only volunteer attention (Madden, Mowry, Gao, Cullen, & Foreman, 2010). A separate group of cancer patients undergoing chemotherapy participating in once-weekly AT sessions demonstrated improvement in fatigue and depression scores as measured by the Hospital Anxiety and Depression Scale and the Brief Fatigue Inventory (Bar-Sela, Atid, Danos, Gabay, & Epelbaum, 2007).

A study conducted at Memorial Sloan-Kettering Cancer Center delivered AT to nine adult patients twice per week while they were in isolation for bone marrow transplant. Results suggest that as it is a nonverbal, metaphorical modality, AT is especially beneficial for patients who need to deal with emotional conflicts and with feelings about life and death in a safe setting (Gabriel et al., 2001). A 1-hour art-making session among blood and bone marrow transplantation recipients decreased the therapy-related symptoms of sluggishness and decreased concentration but did not improve anxiety as compared to control (Lawson et al., 2012).

Among women with breast cancer, AT was shown to decrease depression, anxiety, and somatic symptoms in the postoperative radiotherapy period (Thyme et al., 2009). In women with nonmetastatic breast cancer, AT helped to distinguish cultural understanding about boundaries and provided greater legitimacy to personal interpretations and experience (Oster, Magnusson, Thyme, Lindh, & Astrom, 2007). Other studies support the role of AT in the breast cancer population as a means for assisting women with body change and meaningful expression (Borgmann, 2002; Puig, Lee, Goodwin, & Sherrard, 2006).

Research by Walsh and colleagues suggests a role for AT among family caregivers. Specifically, interventions reduced stress, lowered anxiety, and promoted short-term well-being. Caregivers experienced increased positive communication with patients and health-care providers (Walsh, Martin, & Schmidt, 2004; Walsh, Radcliffe, Castillo, Kumar, & Broschard, 2007). Terminally ill patients (n = 177) from a hospice palliative care unit in Taiwan felt more relaxed in their emotional state, and 53.1% had physical improvement as a result of image appreciation and hands-on painting (Ming-Hwai et al., 2012). A systematic review of 14 papers reporting 12 studies examining the effects of AT on a variety of emotional, physical, social, and spiritual symptoms, as well as global functioning, concluded that AT is used at all stages of the cancer trajectory, most often by women with breast cancer, and that while it is effective in managing symptoms, research in this area is lacking and immature (Wood, Molassiotis, & Payne, 2011).

New to the AT arena is mindfulness-based art therapy (MBAT). This approach couples AT with MBSR in hopes of improving symptom control, processing information, and assigning meaning to the cancer experience. Research is still lacking in this area and additional randomized controlled trials are needed, but at least two trials suggest that MBAT may be of therapeutic benefit among both women and men (Monti et al., 2006; Monti, Gomella, Peterson, & Kunkel, 2007).

**SUPPORT GROUPS** 

Support groups provide information for members, create an emotionally supportive environment of comfort, teach coping skills, help reduce anxiety, and provide a place for people to share common concerns (American Cancer Society, 2012). Groups may be general or cancer-site–specific and may involve patients only or patients and family members. Settings range from hospitals to community-based settings. Early work by Spiegel and colleagues demonstrated that among women with metastatic breast cancer, support groups proved effective for improving coping, decreasing anxiety, and decreasing depression (Spiegel, Bloom, & Yalom, 1981). This finding, along with positive observations about group coping and group bonding, opened the door for future research.

In 1989, 10-year follow-up suggested that women who had participated in the support group lived twice as long as those in the nonsupport control group (Spiegel, Bloom, Kraemer, & Gottheil, 1989). The implication that support groups can improve survival in women with breast cancer has not been confirmed by more recent studies, including an additional study by Spiegel and colleagues (Cunningham et al., 1998; Edelman, Lemon, Bell, & Kidman, 1999; Goodwin et al., 2001; Kissane et al., 2004; Spiegel et al., 2007).

It is widely accepted that support groups enhance QOL and, in an analysis of original studies (including 20 randomized controlled trials) from 14 databases over 20 years, results indicate that participation in support groups is associated with significant improvements in depression, anxiety, illness adaptation, QOL, and marital relationships (Zabalegui, Sanchez, Sanchez, & Juando, 2005). New research is being designed to evaluate the effects of online support groups for cancer patients, but at present, evidence is lacking. It is important to remind patients that many online participants have no medical background and that some online sites do not adhere to expected privacy standards.

**AROMATHERAPY** 

The medicinal use of plants can be traced back to the prehistoric age; interestingly, the use of antibiotics is just over 100 years old. The term aromatherapy was coined by chemist René-Maurice Gattefossé in 1930 in Lyon, France. "Aromatherapy is the controlled and skilled use of essential oils (volatile byproducts of plant metabolism) to maintain health and well-being and to prevent imbalances and illness on the physical, emotional, mental, and spiritual levels, for the good of mankind and preservation of the entire planet" (Cooksley, 2011, p. 43). Essential oils have a variety of properties (see Table 2); are administered in a number of ways (see Table 3); and depending on the oil, have the ability to calm, excite, or balance the individual.

**Table 2:**
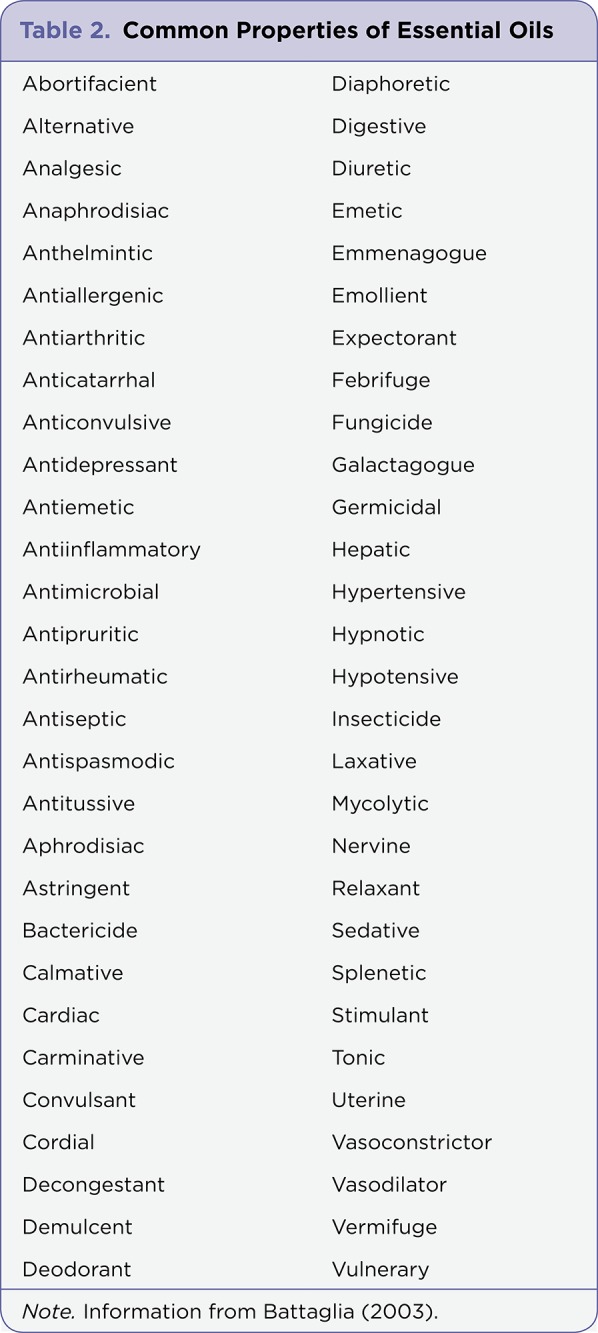
Table 2. Common Properties of Essential Oils

**Table 3:**
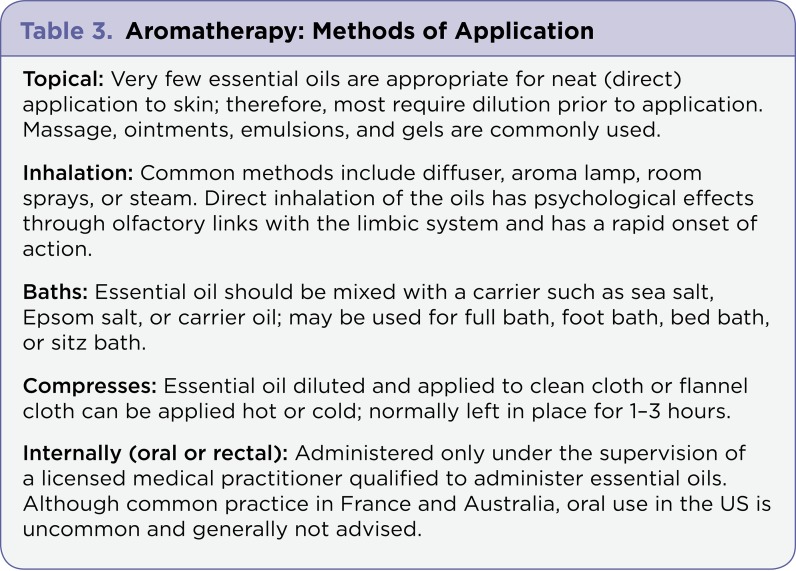
Table 3. Aromatherapy: Methods of Application

Although it is offered in some countries, such as France and Australia, as alternative therapy, current evidence does not support the use of aromatherapy in the oncology setting as an alternative therapy but rather as a complementary treatment modality. Potential benefits from aromatherapy application in the oncology setting include symptom relief, immune support, detoxification, and restoration of the MB balance. Among 17 permanent hospice patients treated with diffused *Lavandula angustifolia* essential oil, a decrease in pulse and blood pressure was noted, along with lowered scores for pain, anxiety, and depression. These scores did not reach statistical significance, but a calmer environment among caregivers was noted at the end of treatment (Louis & Kowalski, 2002). The addition of lavender essential oil to weekly massage in palliative care patients did not appear beneficial, but sleep scores improved in patients treated with massage, with or without the addition of the oil (Soden, Vincent, Craske, Lucas, & Ashley, 2004).

The effects of an aromatherapy inhaler (aromastick) on anxiety, nausea, and sleep disturbance were examined among 160 patients with cancer. A total of 77% of all patients reported deriving at least one benefit from the aromastick. In anxious patients, 65% reported feeling more relaxed and 51% felt less stressed. Nausea was improved in 47% of patients and 55% experienced improved sleep quality (Stringer & Donald, 2011). The evaluation of patient perspectives was not controlled, but the study suggested that additional research utilizing this delivery method is needed. Compared to CBT, aromatherapy massage was at least as effective in all stages of cancer at improving mood, depression, and anxiety scores. There was a nonsignificant trend toward greater improvement in depression with aromatherapy vs. CBT (Serfaty, Wilkinson, Freeman, Mannix, & King, 2012).

In terms of symptom management, there has been extensive research on the application of individual essential oils as treatment for anorexia, anxiety, constipation, depression, fatigue, immune suppression, nausea, secondary infection, and skin impairment. While the majority of findings do support and suggest a role for aromatherapy as a valid complementary modality, the number of well-controlled, statistically powered, randomized controlled trials specific to the oncology setting is limited; additional trials are needed to define an exact role for aromatherapy in specific cancer populations.

## Advanced Practice Implications

Advanced practitioners (APs) are responsible for providing evidence-based, holistic care to patients with a variety of cancer illnesses. Identified barriers to adequate care and open communication with patients include a lack of familiarity with CAM and a dismissive attitude among APs (Cassileth et al., 2007). Despite the increased use of complementary therapies, 38% to 60% of patients with cancer fail to report use to their provider (Deng et al., 2009). Common reasons for not disclosing such use are fear of offending the provider, belief that the topic will be dismissed, and lack of inquiry about patient use on the part of the provider (Cassileth et al., 2007; Deng et al., 2009). Advanced practitioners should communicate an openness to discuss CAM to their patients, and inquiry about CAM use should be documented as a part of every patient encounter.

As suggested earlier, perhaps one reason APs neglect to inquire about or prescribe CAM is a lack of knowledge on the subject. Even though many academic programs are now offering certification in CAM, traditional graduate core curricula for APs have failed to provide a level of education sufficient for ensuring competency outside of traditional medicine; limited direction for incorporating complementary medicine into practice has been given. Advanced practitioners in the academic setting have an opportunity to ensure and demand that information on CAM is a standard part of the educational core curricula.

Incorporation of CAM into clinical practice does not require advanced practice certification or prescriptive authority. Training and licensure can vary from state to state. In the case of the registered nurse, a license to provide CAM services is outlined by each state’s board of nursing via the Nurse Practice Act. For APs, the Advanced Nurse Practice Act and state jurisdiction also provide direction for specialized practice, but they are not specific in terms of CAM (Denner, 2007).

Advanced pracititoners are ethically responsible for maintaining competency when prescribing or recommending CAM. According to the American Holistic Nurses Association (AHNA), "A nurse practicing as a therapist of a specific conventional or CAM therapy must have the education, skills, and credentials ascribed for that therapy" (AHNA, 2012). Certification is one way APs can document that they have met specific competencies in CAM. Advanced Holistic Nurse, Board Certified, certification is available from the American Holistic Nurses’ Credentialing Corporation (AHNCC) for the AP with a Master’s degree from an accredited institution who has completed a minimum of 48 contact hours of continuing education in holistic nursing and has actively practiced as a holistic nurse for a minimum of 1 year full-time or 2,000 hours within the last 5 years part-time (AHNCC, 2012).

All evidence considered suggests additional well-designed, controlled trials with adequate sample size and robust data in the oncology setting are needed to establish an evidence-based comfort zone for routine incorporation of CAM, including MB therapies, into practice. Advanced practitioners are in a prime position to design, implement, and translate integrative research into mainstream practice. In doing so, APs will help to further define the complementary advanced scope of practice and impact state and federal legislation. Additionally, a concrete body of complementary advanced evidence will define standards and limit liability.

## Conclusion

Mind-body therapies, while not new, are becoming increasingly popular complementary therapy options for patients with cancer. Likewise, modern Western medicine is showing renewed interest in the subject, with incorporation of MB therapies into the hospital setting, academia, and clinical training. While some evidence supports mindfulness-based stress reduction, relaxation therapy, cognitive behavioral therapy, hypnosis, biofeedback, music therapy, art therapy, support groups, and aromatherapy as having a place in effective complementary measure for symptom management and improved quality of life, research findings are mixed; additional randomized controlled trials are needed to fully define the optimal use of MB therapies in the oncology setting. Advanced practitioners have a responsibility to guide their patients based on the best available evidence as well as the opportunity to serve as educators, researchers, and integrative oncology leaders.

## References

[1] (2012). American Holistic Nurses Association.. *AHNA*.

[2] (2012). American Holistic Nurses Credentialing Corporation. *AHNCC*.

[3] Al-Mohammed H. (2010). Investigation of breathing maneuvers using free breathing and video biofeedback techniques during radiation therapy treatment for non small cell lung cancer patients. *Journal of Cancer Research and Experimental Oncology*.

[4] (2012). American Art Therapy Association.. *Art therapy: Definition of the profession*.

[5] (2012). American Cancer Society.. *Support groups.*.

[6] (2012). What is music therapy?. *American Music Therapy Association.*.

[7] Appling Susan E, Scarvalone Susan, MacDonald Ryan, McBeth Maureen, Helzlsouer Kathy J (2012). Fatigue in breast cancer survivors: the impact of a mind-body medicine intervention.. *Oncology nursing forum*.

[8] (2008). What is biofeedback?. *Association for Applied Psychophysiology and Biofeedback.*.

[9] Bar-Sela Gil, Atid Lily, Danos Sara, Gabay Naomi, Epelbaum Ron (2007). Art therapy improved depression and influenced fatigue levels in cancer patients on chemotherapy.. *Psycho-oncology*.

[10] Bartlett L, Sloots K, Nowak M, Ho Y-H (2011). Biofeedback therapy for symptoms of bowel dysfunction following surgery for colorectal cancer.. *Techniques in coloproctology*.

[11] Battaglia S. (2003). The International Centre of Holistic Aromatherapy.. *The complete guide to aromatherapy (2nd ed.)*.

[12] Benson H., Klipper M. (1975). *The relaxation response.*.

[13] Borgmann E. (2002). Art therapy with three women diagnosed with cancer. *The Arts in Psychotherapy*.

[14] Bradt J., Dileo C., Grocke D., Magill L. (2011). Music interventions for improving psychological and physical outcomes in cancer patients.. *Cochrane Database of Systematic Reviews*.

[15] Burgio K., Goode P., Urban D., Umlauf M., Locher J., Bueschen A., Redden D. (2006). Preoperative biofeedback assisted behavioral training to decrease post-prostatectomy incontinence: A randomized, controlled trial. *Journal of Urology*.

[16] Burish T G, Jenkins R A (1992). Effectiveness of biofeedback and relaxation training in reducing the side effects of cancer chemotherapy.. *Health psychology : official journal of the Division of Health Psychology, American Psychological Association*.

[17] Campbell Cathy L, Campbell Lisa C (2012). A systematic review of cognitive behavioral interventions in advanced cancer.. *Patient education and counseling*.

[18] Cannici J, Malcolm R, Peek L A (1983). Treatment of insomnia in cancer patients using muscle relaxation training.. *Journal of behavior therapy and experimental psychiatry*.

[19] Carlson Linda E, Speca Michael, Faris Peter, Patel Kamala D (2007). One year pre-post intervention follow-up of psychological, immune, endocrine and blood pressure outcomes of mindfulness-based stress reduction (MBSR) in breast and prostate cancer outpatients.. *Brain, behavior, and immunity*.

[20] Carlson L., Speca M., Patel K., Goodey  E.  (2003). Mindfulness-based stress reduction in relation to quality of life, mood, symptoms of stress, and immune parameters in breast and prostate cancer outpatients. *Psychosomatic Medicine*.

[21] Carlson Linda E, Speca Michael, Patel Kamala D, Goodey Eileen (2004). Mindfulness-based stress reduction in relation to quality of life, mood, symptoms of stress and levels of cortisol, dehydroepiandrosterone sulfate (DHEAS) and melatonin in breast and prostate cancer outpatients.. *Psychoneuroendocrinology*.

[22] Carlson L E, Ursuliak Z, Goodey E, Angen M, Speca M (2001). The effects of a mindfulness meditation-based stress reduction program on mood and symptoms of stress in cancer outpatients: 6-month follow-up.. *Supportive care in cancer : official journal of the Multinational Association of Supportive Care in Cancer*.

[23] Cassileth B., Deng G., Gomez J., Johnstone P., Kumar N., Vickers A. (2007). Complementary therapies and integrative oncology in lung cancer: ACCP evidence-based clinical practice guidelines (2nd ed.). *Chest*.

[24] Cassileth Barrie R, Vickers Andrew J, Magill Lucanne A (2003). Music therapy for mood disturbance during hospitalization for autologous stem cell transplantation: a randomized controlled trial.. *Cancer*.

[25] Clark Michael, Isaacks-Downton Gloria, Wells Nancy, Redlin-Frazier Sheryl, Eck Carol, Hepworth Joseph T, Chakravarthy Bapsi (2006). Use of preferred music to reduce emotional distress and symptom activity during radiation therapy.. *Journal of music therapy*.

[26] Cooksley V., Cooksley V., Pounds L. (2011). The Institute of Integrative Aromatherapy certificate training manual (p. 43). *History of aromatics*.

[27] Cunningham A., Edmonds C., Jenkins G., Pollack H., Lockwood G., Warr D. (1998). A randomized controlled trial of the effects of group psychological therapy on survival in women with metastatic breast cancer.. *Psycho-Oncology*.

[28] Decker T W, Cline-Elsen J, Gallagher M (1992). Relaxation therapy as an adjunct in radiation oncology.. *Journal of clinical psychology*.

[29] Deng Gary E, Frenkel Moshe, Cohen Lorenzo, Cassileth Barrie R, Abrams Donald I, Capodice Jillian L, Courneya Kerry S, Dryden Trish, Hanser Suzanne, Kumar Nagi, Labriola Dan, Wardell Diane W, Sagar Stephen (2009). Evidence-based clinical practice guidelines for integrative oncology: complementary therapies and botanicals.. *Journal of the Society for Integrative Oncology*.

[30] Denner S. (2007). The advanced practice nurse and integration of complementary and alternative medicine: Emerging policy issues.. *Holistic Nursing Practice*.

[31] Edelman S., Lemon J., Bell D., Kidman A. (1999). Effects of group CBT (cognitive behavior therapy) on the survival time of patients with metastatic breast cancer.. *Psycho-Oncology*.

[32] Elkins G., Cheung A., Marcus J., Palamara L., Rajab H.  (2004). Hypnosis to reduce pain in cancer survivors with advanced disease: A prospective study.. *Journal of Cancer Integrative Medicine*.

[33] Elkins Gary, Fisher William, Johnson Aimee (2010). Mind-body therapies in integrative oncology.. *Current treatment options in oncology*.

[34] Elkins Gary, Marcus Joel, Stearns Vered, Perfect Michelle, Rajab M Hasan, Ruud Christopher, Palamara Lynne, Keith Timothy (2008). Randomized trial of a hypnosis intervention for treatment of hot flashes among breast cancer survivors.. *Journal of clinical oncology : official journal of the American Society of Clinical Oncology*.

[35] Ferrer Alejandra J (2007). The effect of live music on decreasing anxiety in patients undergoing chemotherapy treatment.. *Journal of music therapy*.

[36] Foley Elizabeth, Baillie Andrew, Huxter Malcolm, Price Melanie, Sinclair Emma (2010). Mindfulness-based cognitive therapy for individuals whose lives have been affected by cancer: a randomized controlled trial.. *Journal of consulting and clinical psychology*.

[37] Forzoni Silvia, Perez Michela, Martignetti Angelo, Crispino Sergio (2010). Art therapy with cancer patients during chemotherapy sessions: an analysis of the patients’ perception of helpfulness.. *Palliative & supportive care*.

[38] Gabriel B., Bromberg E., Vandenbovenkamp J., Walka P., Kornblith A., Luzzatto P. (2001). Art therapy with adult bone marrow transplant patients in isolation: A pilot study.. *Psycho-Oncology*.

[39] Gallagher Lisa M, Lagman Ruth, Walsh Declan, Davis Mellar P, Legrand Susan B (2006). The clinical effects of music therapy in palliative medicine.. *Supportive care in cancer : official journal of the Multinational Association of Supportive Care in Cancer*.

[40] George Rohini, Chung Theodore D, Vedam Sastry S, Ramakrishnan Viswanathan, Mohan Radhe, Weiss Elisabeth, Keall Paul J (2006). Audio-visual biofeedback for respiratory-gated radiotherapy: impact of audio instruction and audio-visual biofeedback on respiratory-gated radiotherapy.. *International journal of radiation oncology, biology, physics*.

[41] Goodwin P J, Leszcz M, Ennis M, Koopmans J, Vincent L, Guther H, Drysdale E, Hundleby M, Chochinov H M, Navarro M, Speca M, Hunter J (2001). The effect of group psychosocial support on survival in metastatic breast cancer.. *The New England journal of medicine*.

[42] Gordon J. S.  (2008). Mind-body medicine and cancer. *Hematology/Oncology Clinics of North America*.

[43] Hanser Suzanne B, Bauer-Wu Susan, Kubicek Lorrie, Healey Martha, Manola Judith, Hernandez Maria, Bunnell Craig (2006). Effects of a music therapy intervention on quality of life and distress in women with metastatic breast cancer.. *Journal of the Society for Integrative Oncology*.

[44] Haun M, Mainous R O, Looney S W (2001). Effect of music on anxiety of women awaiting breast biopsy.. *Behavioral medicine (Washington, D.C.)*.

[45] Hilliard Russell E (2003). The effects of music therapy on the quality and length of life of people diagnosed with terminal cancer.. *Journal of music therapy*.

[46] Hoffman Caroline J, Ersser Steven J, Hopkinson Jane B, Nicholls Peter G, Harrington Julia E, Thomas Peter W (2012). Effectiveness of mindfulness-based stress reduction in mood, breast- and endocrine-related quality of life, and well-being in stage 0 to III breast cancer: a randomized, controlled trial.. *Journal of clinical oncology : official journal of the American Society of Clinical Oncology*.

[47] Holland J C, Morrow G R, Schmale A, Derogatis L, Stefanek M, Berenson S, Carpenter P J, Breitbart W, Feldstein M (1991). A randomized clinical trial of alprazolam versus progressive muscle relaxation in cancer patients with anxiety and depressive symptoms.. *Journal of clinical oncology : official journal of the American Society of Clinical Oncology*.

[48] Horne-Thompson Anne, Grocke Denise (2008). The effect of music therapy on anxiety in patients who are terminally ill.. *Journal of palliative medicine*.

[49] Jacknow D S, Tschann J M, Link M P, Boyce W T (1994). Hypnosis in the prevention of chemotherapy-related nausea and vomiting in children: a prospective study.. *Journal of developmental and behavioral pediatrics : JDBP*.

[50] Jia Libin (2012). Cancer complementary and alternative medicine research at the US National Cancer Institute.. *Chinese journal of integrative medicine*.

[51] Kaliyaperumal R., Subash J. (2010). Effect of music therapy for patients with cancer pain. *International Journal of Biological & Medical Research*.

[52] Kang Duck-Hee, McArdle Traci, Park Na-Jin, Weaver Michael T, Smith Barbara, Carpenter John (2011). Dose effects of relaxation practice on immune responses in women newly diagnosed with breast cancer: an exploratory study.. *Oncology nursing forum*.

[53] Kim Keum Soon, Lee So Woo, Choe Myoung Ae, Yi Myung Sun, Choi Smi, Kwon So-Hi (2005). [Effects of abdominal breathing training using biofeedback on stress, immune response and quality of life in patients with a mastectomy for breast cancer].. *Taehan Kanho Hakhoe chi*.

[54] Kim Kyung Ho, Yu Chang Sik, Yoon Yong Sik, Yoon Sang Nam, Lim Seok-Byung, Kim Jin Cheon (2011). Effectiveness of biofeedback therapy in the treatment of anterior resection syndrome after rectal cancer surgery.. *Diseases of the colon and rectum*.

[55] Kissane David W, Love Anthony, Hatton Allison, Bloch Sidney, Smith Graeme, Clarke David M, Miach Patricia, Ikin Jill, Ranieri Nadia, Snyder Raymond D (2004). Effect of cognitive-existential group therapy on survival in early-stage breast cancer.. *Journal of clinical oncology : official journal of the American Society of Clinical Oncology*.

[56] Kwekkeboom Kristine L, Abbott-Anderson Kristen, Cherwin Catherine, Roiland Rachel, Serlin Ronald C, Ward Sandra E (2012). Pilot randomized controlled trial of a patient-controlled cognitive-behavioral intervention for the pain, fatigue, and sleep disturbance symptom cluster in cancer.. *Journal of pain and symptom management*.

[57] Kwekkeboom Kristine L, Cherwin Catherine H, Lee Jun W, Wanta Britt (2010). Mind-body treatments for the pain-fatigue-sleep disturbance symptom cluster in persons with cancer.. *Journal of pain and symptom management*.

[58] Kwekkeboom Kristine L, Hau Hannah, Wanta Britt, Bumpus Molly (2008). Patients’ perceptions of the effectiveness of guided imagery and progressive muscle relaxation interventions used for cancer pain.. *Complementary therapies in clinical practice*.

[59] Lang E V, Benotsch E G, Fick L J, Lutgendorf S, Berbaum M L, Berbaum K S, Logan H, Spiegel D (2000). Adjunctive non-pharmacological analgesia for invasive medical procedures: a randomised trial.. *Lancet*.

[60] Lang Elvira V, Berbaum Kevin S, Faintuch Salomao, Hatsiopoulou Olga, Halsey Noami, Li Xinyu, Berbaum Michael L, Laser Eleanor, Baum Janet (2006). Adjunctive self-hypnotic relaxation for outpatient medical procedures: a prospective randomized trial with women undergoing large core breast biopsy.. *Pain*.

[61] Lang Elvira V, Berbaum Kevin S, Pauker Stephen G, Faintuch Salomao, Salazar Gloria M, Lutgendorf Susan, Laser Eleanor, Logan Henrietta, Spiegel David (2008). Beneficial effects of hypnosis and adverse effects of empathic attention during percutaneous tumor treatment: when being nice does not suffice.. *Journal of vascular and interventional radiology : JVIR*.

[62] Lawson Lisa Mische, Williams Phoebe, Glennon Cathy, Carithers Kendall, Schnabel Erin, Andrejack Amy, Wright Nicole (2012). Effect of art making on cancer-related symptoms of blood and marrow transplantation recipients.. *Oncology nursing forum*.

[63] Lee Richard T, Hlubocky Fay J, Hu Je-Jen, Stafford Randall S, Daugherty Christopher K (2008). An international pilot study of oncology physicians’ opinions and practices on Complementary and Alternative Medicine (CAM).. *Integrative cancer therapies*.

[64] Li Xiao-Mei, Yan Hong, Zhou Kai-Na, Dang Shao-Nong, Wang Duo-Lao, Zhang Yin-Ping (2011). Effects of music therapy on pain among female breast cancer patients after radical mastectomy: results from a randomized controlled trial.. *Breast cancer research and treatment*.

[65] Lin Mei-Feng, Hsieh Ya-Ju, Hsu Yu-Yun, Fetzer Susan, Hsu Mei-Chi (2011). A randomised controlled trial of the effect of music therapy and verbal relaxation on chemotherapy-induced anxiety.. *Journal of clinical nursing*.

[66] Liossi C, Hatira P (1999). Clinical hypnosis versus cognitive behavioral training for pain management with pediatric cancer patients undergoing bone marrow aspirations.. *The International journal of clinical and experimental hypnosis*.

[67] Lorentz M. (2006). Stress and psychoneuroimmunology. *Alternative Journal of Nursing*.

[68] Louis M., Kowalski S. (2002). Use of aromatherapy with hospice patients to decrease pain, anxiety, and depression and to promote an increased sense of well-being.. *American Journal of Hospice & Palliative Care*.

[69] Mao Jun James, Palmer Christina Shearer, Healy Kaitlin Elizabeth, Desai Krupali, Amsterdam Jay (2011). Complementary and alternative medicine use among cancer survivors: a population-based study.. *Journal of cancer survivorship : research and practice*.

[70] Matchim Yaowarat, Armer Jane M, Stewart Bob R (2011). Mindfulness-based stress reduction among breast cancer survivors: a literature review and discussion.. *Oncology nursing forum*.

[71] Lin Ming-Hwai, Moh Shwu-Lan, Kuo Yu-Cheng, Wu Pin-Yuan, Lin Chiung-Ling, Tsai Mei-Hui, Chen Tzeng-Ji, Hwang Shinn-Jang (2012). Art therapy for terminal cancer patients in a hospice palliative care unit in Taiwan.. *Palliative & supportive care*.

[72] Montgomery Guy H, Bovbjerg Dana H, Schnur Julie B, David Daniel, Goldfarb Alisan, Weltz Christina R, Schechter Clyde, Graff-Zivin Joshua, Tatrow Kristin, Price Donald D, Silverstein Jeffrey H (2007). A randomized clinical trial of a brief hypnosis intervention to control side effects in breast surgery patients.. *Journal of the National Cancer Institute*.

[73] Montgomery Guy H, Hallquist Michael N, Schnur Julie B, David Daniel, Silverstein Jeffrey H, Bovbjerg Dana H (2010). Mediators of a brief hypnosis intervention to control side effects in breast surgery patients: response expectancies and emotional distress.. *Journal of consulting and clinical psychology*.

[74] Montgomery Guy H, Weltz Christina R, Seltz Megan, Bovbjerg Dana H (2002). Brief presurgery hypnosis reduces distress and pain in excisional breast biopsy patients.. *The International journal of clinical and experimental hypnosis*.

[75] Monti D., Gomella L., Peterson C., Kunkel E. (2007). Preliminary results from a novel psychosocial program for men with prostate cancer.. *Abstract presented at the 2007 Prostate Cancer Symposium, Orlando, Florida*.

[76] Monti Daniel A, Peterson Caroline, Kunkel Elisabeth J Shakin, Hauck Walter W, Pequignot Edward, Rhodes Lora, Brainard George C (2006). A randomized, controlled trial of mindfulness-based art therapy (MBAT) for women with cancer.. *Psycho-oncology*.

[77] Monti Daniel A, Sufian Meryl, Peterson Caroline (2008). Potential role of mind-body therapies in cancer survivorship.. *Cancer*.

[78] Morrow G R, Morrell C (1982). Behavioral treatment for the anticipatory nausea and vomiting induced by cancer chemotherapy.. *The New England journal of medicine*.

[79] Musial Frauke, Büssing Arndt, Heusser Peter, Choi Kyung-Eun, Ostermann Thomas (2011). Mindfulness-based stress reduction for integrative cancer care: a summary of evidence.. *Forschende Komplementärmedizin (2006)*.

[80] National Institutes of Health Technology Assessment Panel. (1996). Integration of behavioral and relaxation approaches into the treatment of chronic pain and insomnia. NIH technology assessment panel on integration of behavioral and relaxation approaches into the treatment of chronic pain and insomnia.. *Journal of the American Medical Association*.

[81] NCCAM (2012). * National Center for Complementary and Alternative Medicine.*.

[82] Nguyen T., Nilsson S., Hellstrom A., Bengtson A. (2010). Music therapy to reduce pain and anxiety in children with cancer undergoing lumbar puncture: A randomized clinical trial.. *Journal of Pediatric Oncology Nursing*.

[83] Oster I., Magnusson E., Thyme K., Lindh J., Astrom S. (2007). Art therapy for women with breast cancer: The therapeutic consequences of boundary strengthening.. *The Arts in Psychotherapy*.

[84] Plaskota Marek, Lucas Caroline, Evans Rosie, Cook Karen, Pizzoferro Kathleen, Saini Treena (2012). A hypnotherapy intervention for the treatment of anxiety in patients with cancer receiving palliative care.. *International journal of palliative nursing*.

[85] Puig A., Lee S., Goodwin L., Sherrard P. (2006). The efficacy of creative arts therapies to enhance emotional expression, spirituality, and psychological well being of newly diagnosed stage I and stage II breast cancer patients: A preliminary study. *The Arts in Psychotherapy*.

[86] Rajasekaran M, Edmonds P M, Higginson I L (2005). Systematic review of hypnotherapy for treating symptoms in terminally ill adult cancer patients.. *Palliative medicine*.

[87] Sabo C E, Michael S R (1996). The influence of personal message with music on anxiety and side effects associated with chemotherapy.. *Cancer nursing*.

[88] Schnur Julie B, David Daniel, Kangas Maria, Green Sheryl, Bovbjerg Dana H, Montgomery Guy H (2009). A randomized trial of a cognitive-behavioral therapy and hypnosis intervention on positive and negative affect during breast cancer radiotherapy.. *Journal of clinical psychology*.

[89] Serfaty M, Wilkinson S, Freeman C, Mannix K, King M (2012). The ToT study: helping with Touch or Talk (ToT): a pilot randomised controlled trial to examine the clinical effectiveness of aromatherapy massage versus cognitive behaviour therapy for emotional distress in patients in cancer/palliative care.. *Psycho-oncology*.

[90] Shennan Christina, Payne Sheila, Fenlon Deborah (2011). What is the evidence for the use of mindfulness-based interventions in cancer care? A review.. *Psycho-oncology*.

[91] Soden Katie, Vincent Karen, Craske Stephen, Lucas Caroline, Ashley Sue (2004). A randomized controlled trial of aromatherapy massage in a hospice setting.. *Palliative medicine*.

[92] Speca M., Carlson L., Goodey E., Angen M. (2000). A randomized, wait-list controlled clinical trial: The effect of a mindfulness meditation-based stress reduction program on mood and symptoms of stress in cancer outpatients. *Psychosomatic Medicine*.

[93] Spiegel D, Bloom J R (1983). Group therapy and hypnosis reduce metastatic breast carcinoma pain.. *Psychosomatic medicine*.

[94] Spiegel D, Bloom J R, Kraemer H C, Gottheil E (1989). Effect of psychosocial treatment on survival of patients with metastatic breast cancer.. *Lancet*.

[95] Spiegel D, Bloom J R, Yalom I (1981). Group support for patients with metastatic cancer. A randomized outcome study.. *Archives of general psychiatry*.

[96] Spiegel David, Butler Lisa D, Giese-Davis Janine, Koopman Cheryl, Miller Elaine, DiMiceli Sue, Classen Catherine C, Fobair Patricia, Carlson Robert W, Kraemer Helena C (2007). Effects of supportive-expressive group therapy on survival of patients with metastatic breast cancer: a randomized prospective trial.. *Cancer*.

[97] Stringer Jacqui, Donald Graeme (2011). Aromasticks in cancer care: an innovation not to be sniffed at.. *Complementary therapies in clinical practice*.

[98] Stringer Jacqui, Donald Graeme (2011). Aromasticks in cancer care: an innovation not to be sniffed at.. *Complementary therapies in clinical practice*.

[99] Syrjala K L, Cummings C, Donaldson G W (1992). Hypnosis or cognitive behavioral training for the reduction of pain and nausea during cancer treatment: a controlled clinical trial.. *Pain*.

[100] Thyme Karin Egberg, Sundin Eva C, Wiberg Britt, Oster Inger, Aström Sture, Lindh Jack (2009). Individual brief art therapy can be helpful for women with breast cancer: a randomized controlled clinical study.. *Palliative & supportive care*.

[101] Tienforti Daniele, Sacco Emilio, Marangi Francesco, D’Addessi Alessandro, Racioppi Marco, Gulino Gaetano, Pinto Francesco, Totaro Angelo, D’Agostino Daniele, Bassi Pierfrancesco (2012). Efficacy of an assisted low-intensity programme of perioperative pelvic floor muscle training in improving the recovery of continence after radical prostatectomy: a randomized controlled trial.. *BJU international*.

[102] Tsai P., Chen P., Lai Y., Lee M., Lin C. (2007). Effects of electromyography biofeedback-assisted relaxation on pain in patients with advanced cancer in a palliative care unit.. *Cancer Nursing*.

[103] Verkerk  R. (2009). Can the failing western medical paradigm be shifted using the principle of sustainability?. *Australasian College of Nutritional & Environmental Medicine*.

[104] Walsh Sandra M, Martin Susan Culpepper, Schmidt Lee A (2004). Testing the efficacy of a creative-arts intervention with family caregivers of patients with cancer.. *Journal of nursing scholarship : an official publication of Sigma Theta Tau International Honor Society of Nursing / Sigma Theta Tau*.

[105] Walsh S., Radcliffe R., Castillo L.,, Kumar  A., Broschard D. (2007). A pilot study to test the effects of art-making classes for family caregivers of patients with cancer.. *Oncology Nursing Forum*.

[106] Witek-Janusek Linda, Albuquerque Kevin, Chroniak Karen Rambo, Chroniak Christopher, Durazo-Arvizu Ramon, Mathews Herbert L (2008). Effect of mindfulness based stress reduction on immune function, quality of life and coping in women newly diagnosed with early stage breast cancer.. *Brain, behavior, and immunity*.

[107] Wood Michele J M, Molassiotis Alexander, Payne Sheila (2011). What research evidence is there for the use of art therapy in the management of symptoms in adults with cancer? A systematic review.. *Psycho-oncology*.

[108] Yildirim Yasemin, Parlar Serap, Eyigor Sibel, Sertoz Ozen O, Eyigor Can, Fadiloglu Cicek, Uyar Meltem (2010). An analysis of nursing and medical students’ attitudes towards and knowledge of complementary and alternative medicine (CAM).. *Journal of clinical nursing*.

[109] Zabalegui Adelaida, Sanchez Susana, Sanchez Pablo D, Juando Clara (2005). Nursing and cancer support groups.. *Journal of advanced nursing*.

[110] Zaza Christine, Sellick Scott M, Hillier Loretta M (2005). Coping with cancer: what do patients do.. *Journal of psychosocial oncology*.

[111] Zeltzer L K, Dolgin M J, LeBaron S, LeBaron C (1991). A randomized, controlled study of behavioral intervention for chemotherapy distress in children with cancer.. *Pediatrics*.

[112] Zhou Kai-na, Li Xiao-mei, Yan Hong, Dang Shao-nong, Wang Duo-lao (2011). Effects of music therapy on depression and duration of hospital stay of breast cancer patients after radical mastectomy.. *Chinese medical journal*.

